# Metacognitive Deficiency in a Perceptual but Not a Memory Task in Methadone Maintenance Patients

**DOI:** 10.1038/s41598-017-06707-w

**Published:** 2017-08-01

**Authors:** Saeedeh Sadeghi, Hamed Ekhtiari, Bahador Bahrami, Majid Nili Ahmadabadi

**Affiliations:** 1 Cognitive Systems Laboratory, School of Electrical and Computer Engineering, University of Tehran, Tehran, Iran; 2 Neurocognitive Laboratory, Iranian National Center for Addiction Studies, Tehran University of Medical Sciences, Tehran, Iran; 3 UCL Institute of Cognitive Neuroscience, University College London, London, United Kingdom; 4 School of Cognitive Science, Institute for Research in Fundamental Sciences, Tehran, Iran

## Abstract

Drug addiction has been associated with lack of insight into one’s own abilities. However, the scope of metacognition impairment among drug users in general and opiate dependent individuals in particular is not fully understood. Investigating the impairments of metacognitive ability in Substance Dependent Individuals (SDIs) in different cognitive tasks could contribute to the ongoing debate over whether metacognition has domain-general or domain-specific neural substrates. We compared metacognitive self-monitoring ability of a group of SDIs during methadone maintenance treatment (n = 23) with a control group (n = 24) in a memory and a visual perceptual task. Post decision self judgements of probability of correct choice were obtained through trial by trial confidence ratings and were used to compute metacognitive ability. Results showed that despite comparable first order performance in the perceptual task, SDIs had lower perceptual metacognition than the control group. However, although SDIs had poorer memory performance, their metacognitive judgements in the memory task were as accurate as the control group. While it is commonly believed that addiction causes pervasive impairment in cognitive functions, including metacognitive ability, we observed that the impairment was only significant in one specific task, the perceptual task, but not in the memory task.

## Introduction

We all make mistakes, from time to time. And we wouldn’t have, had we known that they are mistakes. However, most of us are somewhat aware of the probability that our decision might be inaccurate. The ability to introspect and monitor one’s own performance is called metacognitive ability. Impairments in metacognition have been observed in several neurological diseases (reviewed in David, *et al*.^1^ and Ullsperger^2^) such as Alzheimer’s disease^3^, schizophrenia^4^, early phase psychosis^5^, traumatic brain injury^6^, anterior prefrontal lesions^7^, as well as in pathological gambling^8^. Patients with metacognitive deficiencies may have a misguided feeling of knowing about things they are completely ignorant about or imagine that they have done actions that they never took in reality. Accordingly, these metacognitive deficits are known as disturbances of “insight”. Patients lacking insight might be unaware of the severity of their disease or dispute their need for therapy and medical help. Here we ask if metacognitive deficiencies can be found in disorders of substance dependence^9^.

Substance dependence is characterized by persistent drug use despite negative consequences. Substance Dependent Individuals (SDIs) are impaired, among other things, in a wide range of cognitive functions; however, they seldom have a realistic impression of these problems. Lack of insight is often one of the greatest challenges for the treatment of drug addiction. These patients tend to underestimate their addiction severity^10^ and are unconsciously biased to drug-related cues^11^. Defective self-awareness in SDIs is not limited to behaviors or thoughts directly related to drugs, but it also includes non-drug related cognitive tasks. For instance, chronic cannabis users showed a significant deficit in awareness of commission errors in a go/no go task^12^. Also, individuals with cocaine use disorder had weaker self-monitoring performance in a visuo-perceptual task^13^. However, whether such metacognitive deficits are indicative of a generalized failure of self-monitoring in substance dependence or alternatively, are specific to the cognitive task under study is unknown. Here we sought to replicate self-monitoring deficits previously reported in SDIs and ask if such deficits are general or depend on the specific cognitive task under study. The answer to this question can have important consequences for the controversial issue of domain specificity of metacognition^14–16^.

Numerous methods have been developed to measure metacognitive sensitivity quantitatively across individuals and domains. These methods invariably involve making decisions and reporting the choice uncertainty by describing the vividness of one’s experience^17^, rating confidence^18, 19^ or making a financial wager^20^. Here we used the confidence rating paradigm to evaluate the metacognitive ability.

We compared the metacognitive ability of a group of SDIs with a matched control group in two tasks involving memory or perception. Specifically, our SDIs were people with opiate dependence under methadone maintenance treatment. Metacognition was examined in a word recognition memory task with controlled words’ imageability ratings and a visual search for contrast oddball task. Each trial of both tasks consisted of a 2-Alternetaive Forced Choice (2-AFC) question plus a confidence rating about the accuracy of the response. Meta-d′, a measure based on Signal Detection Theory^21^, was used to quantify metacognition in both tasks^22, 23^.

We asked if metacognitive ability in substance dependent individuals were any different from healthy population in the two tasks involving memory or perception. Our results showed that metacognition is significantly impaired specifically in one task (perceptual) but not in the other in our SDIs group.

## Results

### General analysis

For two of the SDIs and one of the control participants, the staircase did not converge to the predefined 71% level of accuracy in the perceptual task. These participants had accuracy level lower than mean-8*SD of the whole population. They were excluded from further analysis. During debriefing after the task completion, these participants stated that they did recognize the spatial location of the odd Gabor in most trials, but had difficulty in discriminating the two stimulus displays as two separate intervals. For all other participants (N = 47: 23 SDI and 24 control) staircase successfully restricted variations of individual performance (mean accuracy 71%, SD 1%, range 68–73%). Therefore, groups did not differ in perceptual accuracy as expected (t(45) = −0.84, p = 0.4). Contrast threshold varied considerably among participants (range 0.043–0.47, SD 0.08). However, memory accuracy ranged widely (53–84%, SD 7.6%) since there was no systematic method to control memory performance.

To verify that introspective ability was not contaminated by basic task performance we examined the correlation between metacognitive ability (m-ratio) and type 1 task performance (d′). Combining data of all participants in both groups, no such correlation was found in memory (r = −0.19, p = 0.21) nor visual perception (r = 0.07, p = 0.66).

**Figure 1:**
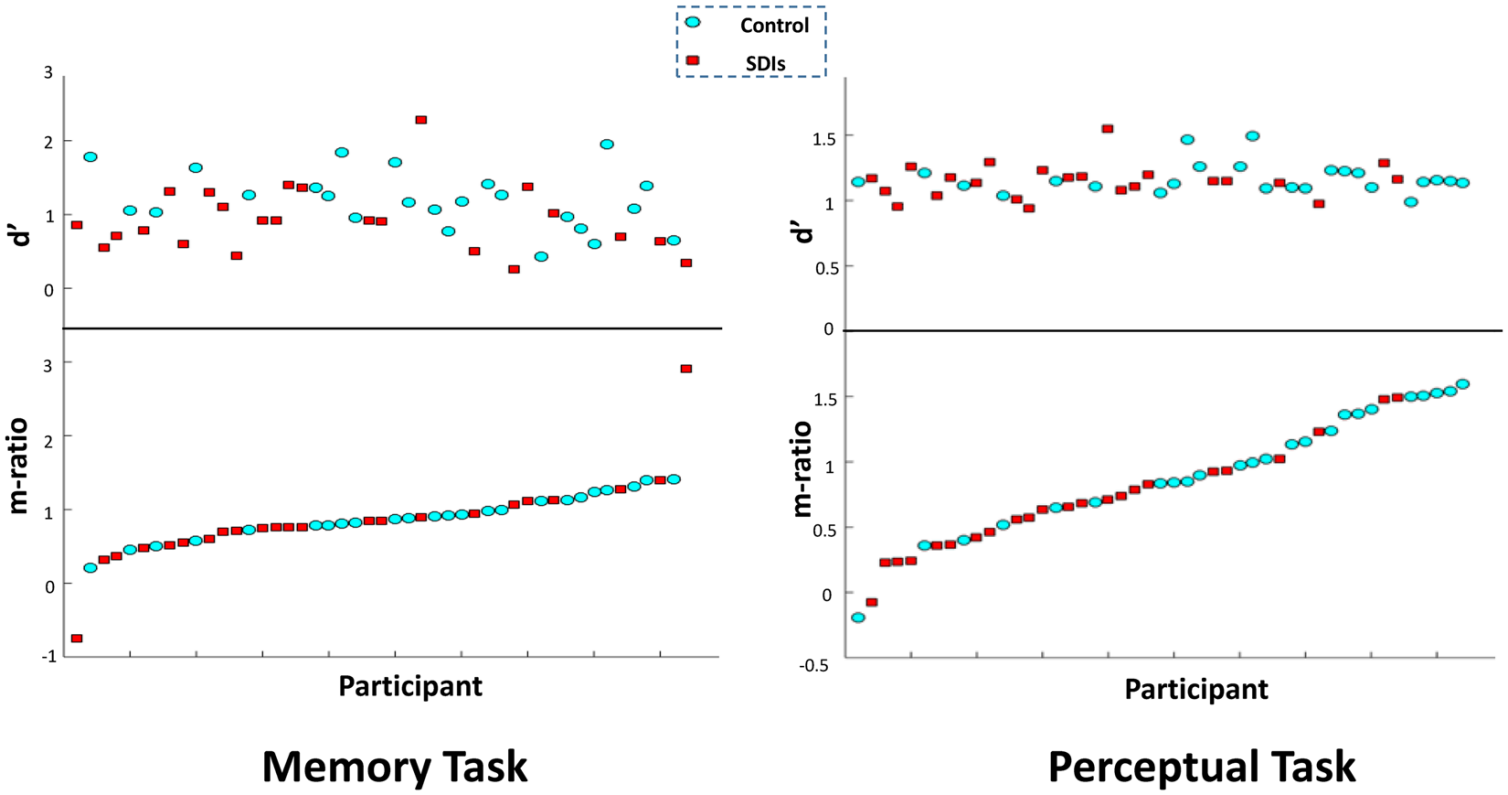
Metacognition (m-ratio) and type 1 performance (d′) in memory (left) and perceptual (right) tasks. The 47 participants (24 Control + 23 SDI) are sorted by their m-ratio in task.

Figure 1 illustrates metacognition and type 1 performance (d′) of participants. One participant in the memory task and two other participants in the perceptual task had below zero m-ratio. All three of them had negative meta-d′ and positive d′ which resulted in negative m-ratio. It means that they were worse than a random confidence generator in rating their confidence, when their responses were modeled by the type 2 SDT. However they were better than random in their 2-AFC answers when modeled by type 1 SDT. We kept these participants in our analysis to avoid any biases in selectively removing any data, though excluding them did not affect our main findings.

Consistent with a previous study^24^ no significant correlation was observed between metacognition in the memory and perceptual tasks in either of the groups (SDI: r = −0.01, p = 0.97; Control: r = 0.34, p = 0.10) nor when all data was pulled across the two groups (r = 0.12, p = 0.42).

### Performance, Metacognitive ability and confidence

The main behavioral measures of the two groups are listed in Table 1. Comparing objective task performances, SDIs and control participants were not significantly different in perceptual acuity (contrast threshold, t(34.4) = 1.59 p = 0.12) or perceptual task performance (d′, t(45) = −0.55, p = 0.59). In the memory task however, the SDIs had a worse performance in recognition of words (d′, t(45) = −2.17, p = 0.04)).

**Table 1:** Behavioral measures for the SDI and Control participants. Mean scores and Standard Deviation (SD) are presented.

Task		Mean (SD)	
		SDI	Control
**Perceptual**	Contrast Threshold	0.19 (0.09)	0.16 (0.05)
	Accuracy	0.71 (0.01)	0.71 (0.01)
	d′	1.15 (0.13)	1.17 (0.12)
	Mean Confidence	4.68 (0.95)	4.74 (0.86)
	M-ratio	0.67 (0.38)	1.01 (0.44)
**Memory**	d′	0.92 (0.44)	1.19 (0.40)
	Mean Confidence	4.63 (0.72)	4.94 (0.71)
	Unequal Variance M-ratio	0.82 (0.61)	0.93 (0.30)
	Equal Variance M-ratio	0.85 (0.66)	0.85 (0.39)

A mixed effect ANOVA was conducted to investigate the effect of task or group on mean confidence. Results showed no significant main effect of task (F(1,45) = 0.45, p = 0.51, η^2^ = 0.010) nor group (F(1,45) = 0.74, p = 0.40, η^2^ = 0.016). In other words, mean confidence rating across trials was not different between the SDIs and control participants in either of the tasks.

**Figure 2:**
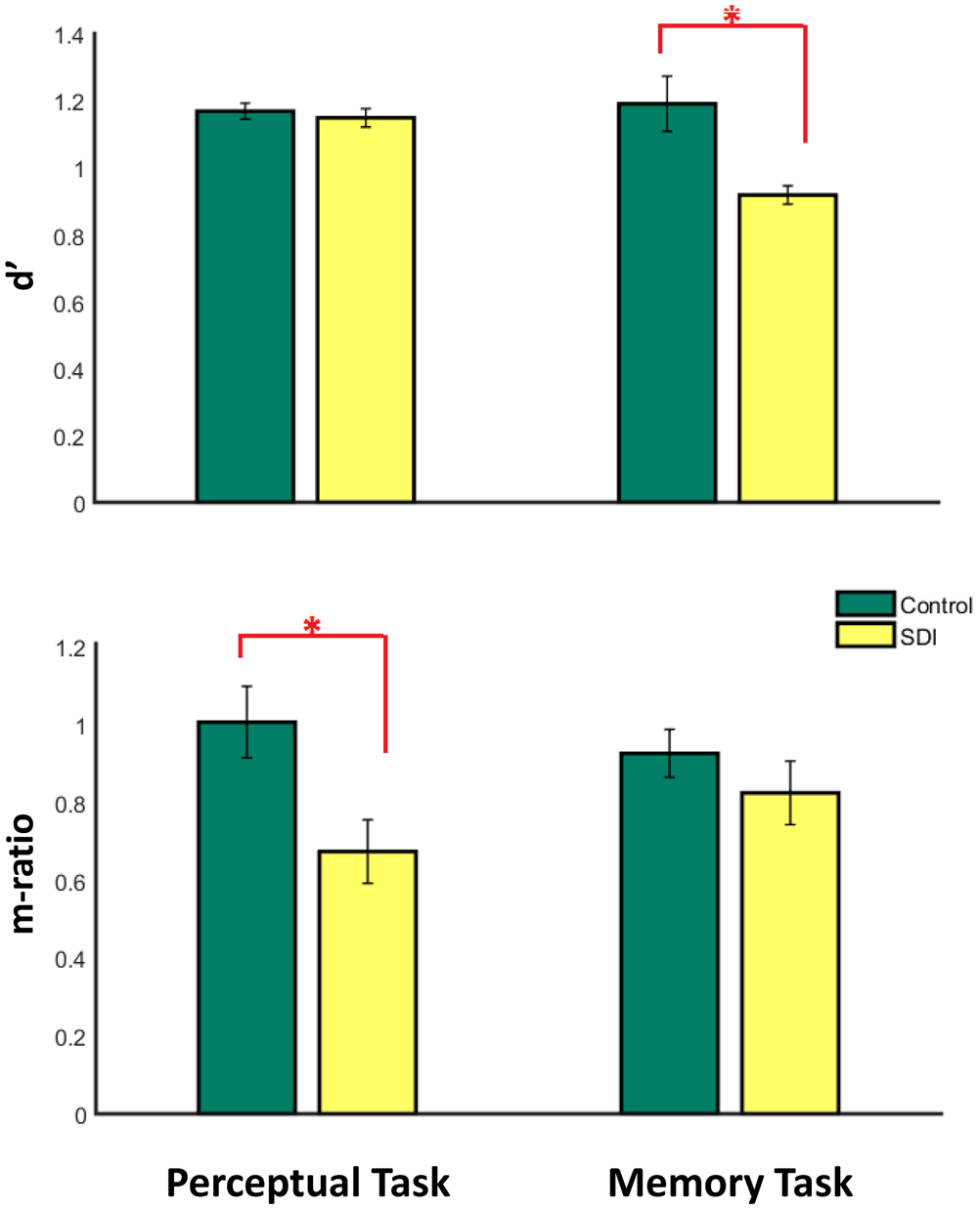
d′ (top) and m-ratio (bottom) in perceptual (left) and memory (right) tasks. SDIs are significantly weaker in memory task type 1 sensitivity and perceptual task metacognitive sensitivity. Perceptual task type 1 sensitivity and memory task metacognition do not significantly differ between the two groups. *Means p < 0.05.

Despite comparable mean confidence ratings and objective performance in the perceptual task, perceptual task metacognitive ability was lower for the SDIs compared to the control participants (M-ratio, t(45) = −2.70, p < = 0.01). In contrast, in the memory task, although SDIs had poorer basic performance in remembering words, they were as accurate as the healthy controls in memory metacognition (unequal variance M-ratio, t(45) = −0.72, p > 0.48). Repeating the analysis with equal variance assumption for M-ratio (meta-d′/d′) did not change this result (equal variance M-ratio, t(45) = −0.03 p = 0.98). Assuming that equal-variance m-ratio in the visual task and unequal-variance m-ratio in the memory task have similar units, there was no significant Group × Task interaction effect on metacognition (mixed effect ANOVA, F(1,45) = 1.66, p = 0.20, η^2^ = 0.034). However, when equal-variance measure was calculated for metacognition in both tasks, there was a marginally significant interaction between group and task on metacognition (mixed effect ANOVA, F(1,45) = 3.16, p = 0.08, η^2^ = 0.066).The main results from testing our key hypotheses are summarized in Fig. 2.

### Response time and Confidence Time

We also analyzed the response and confidence times as presented in Table 2. In the visual perceptual task, the Response time (RT) was defined as the time between the 2^nd^ interval termination and the key press indicating the choice in the 2-alternative response. RT in the memory task was the time interval since onset of the testing word on the screen until pressing the old/new response keys. Confidence time (CT) in both tasks was defined as time after 1^st^ response until key press indicating the confidence rating. In the perceptual task, there was a marginally significant difference between the two groups in average RT (t(31.8) = 1.96, p = 0.06) and a significant difference in average perceptual CT (t(24.3) = 2.44, p = 0.02), indicating that SDIs paused a longer time before reporting their response and confidence. In the memory task, however, no significant difference was found between SDIs and Control participants in average CT (t(45) = 1.48, p = 0.14) or RT (t(45) = 1.32, p = 0.20).

**Table 2:** Mean Response Time (RT) and mean Confidence Time (CT) for SDI and Control groups.

Task		SDI	Control
**Perceptual**	Mean RT	1358 (573)	1099 (283)
	Mean CT	831 (613)	510 (143)
**Memory**	Mean RT	2146 (518)	1964(425)
	Mean CT	683 (226)	577 (262)

### Imageability

Thus far, all 200 trials of the memory test were included in memory analysis; though, as mentioned earlier, memory words were labeled as either high or low imageable. In order to examine the effect of word imageability on metacognitive ability in the memory task, metacognition (meta-da/da) was calculated independently for the high and low imageable words. A mixed effect ANOVA revealed no significant interaction between imageability (high/low) and group (SDI/control) on memory task metacognition (F(1,45) = 0.16, p = 0.69, η^2^ = 0.003) and no main effect of imageability was found(F(1,45) = 0.16, p = 0.84, η^2^ = 0.001). Moreover, there was no correlation between metacognition of high and low imageable words in either of the groups (SDI: r = 0.36, p = 0.11; Control: r = −0.09, p = 0.69).

## Discussion

The clinical indicators of self-awareness impairment in SDIs have been described previously in the literature (see, e.g., Goldstein, *et al*.^9^ and Moeller and Goldstein^25^). However, the precise scope of such impairment is not yet fully understood. We compared self-monitoring metacognitive ability of a group of methadone maintenance patients with a matched control group in a memory and a perceptual task. Our results revealed that compared to healthy controls, in patients with history of opiate dependence first order performance accuracy and second order metacognitive ability for vision and memory dissociated in opposite directions. The SDIs had defective self-monitoring ability in visual perception, despite intact perceptual acuity. In contrast, they were as good as the control group in memory metacognitive ability, even though their first order memory performance was weaker.

To our knowledge this is the first study to examine word recognition memory metacognitive ability in the SDIs using meta-d′ method, which is an estimate of pure metacognition independent of decision bias, confidence bias or first order performance. Several previous studies on SDIs’ word recognition memory (for instance Mintzer and Stitzer^26^ and Le Berre, *et al*.^27^) used assessment methods that, recent works^28^ suggest might be confounded by bias or first order performance. It is worth noting that the SDIs’ bias or first order accuracy were not equal to healthy individuals in those studies. Having this in mind, their methods prevented from comparison of pure metacognition between SDIs and control. Here, the SDIs were weaker in the first order memory performance and the effect of task difficulty in the metacognition estimation was eliminated using the M-ratio measure.

An ongoing debate about neuronal substrates of metacognition has focused on whether metacognitive ability is domain specific or domain general^7, 14–16^. If a person is good at monitoring his memory capacity, is he also good at monitoring his perceptual ability? Is metacognitive ability in different domains dependent on the same or different mechanistic (e.g. neural) substrates? These questions have recently been investigated intensively but conflicting evidence from different studies have prevented definitive conclusions. Behavioral correlation between perceptual and memory metacognitive abilities have been observed^16^, but evidence for their independence has also been provided^15^. Studies of metacognitive ability in neurological patients have shed some light on these issues^7^. If metacognition involved a unique general neural pathway integrating all tasks, patients should either be impaired or intact in all tasks. Here we observed that the SDIs have significantly impaired metacognition in one task (visual perceptual task) but not in the other (memory task). Therefore, our results lend some support to the hypothesis that separate neural substrates for memory and visual metacognitive abilities might exist. In this view, isolated impairment of visual metacognition in SDIs could indicate some specificity of metacognitive processes in the domains of memory and perception. However, one must bear in mind that the perception vs memory dichotomy inferred here, is by virtue of the limitations of empirical experimentation, based on tasks that are different in more than just one cognitive domain (for example, the word recognition memory task involves language systems). Future research focused on testing the stability and replicability of metacognitive impairment in Opioid dependence will be critical for strengthening the domain specificity claims proposed here.

Another concern of this study was to examine the impact of word imageability on metacognitive ability in the memory task, which has not been investigated before. In several previous experiments with similar memory task to ours for assessing metamemory, words with high imageability were used (e.g. McCurdy, *et al*.^16^), while in other experiments no control over words’ imageability was taken (e.g. Baird, *et al*.^24^). Here we chose the memory words such that half were categorized as high imageable and half as low imageable. Separately calculating metacognitive ability in remembering high and low imageable words for each person, there was no correlation between the metacognition for the two divisions of words. Lack of correlation between metacognitive ability for high and low imageable words could signal the complexity of memory metacognitive processes and the potential impact of word imageability on memory metacognition. Intra-individual instability of memory metacognitive ability has been reported previously^29, 30^. On the other hand, one possibility is that increasing number of trials might lead to more stable metamemory results^29^. In our experiment, number of trials used for estimating high or low imageable words’ type 2 sensitivity is only 100, half of the total number of trials in the memory task. Lack of correlation between high and low imageable words’ recognition metacognition could originate from a true effect of imageability on memory metacognition, or be due to the noise of metacognition estimation. Further experiments with higher number of trials are suggested to achieve a more robust conclusion.

Our results are unable to identify the specific mechanism for SDIs’ metacognitive impairment in the visual but not the memory domain. Studying the neural underpinnings dissociating perceptual and memory task metacognitive performances in SDIs is an interesting future research. Furthermore, understanding how the findings from the laboratory-based controlled experiments on metacognitive ability could be implicated in everyday life is another important avenue for future research. In the real world, one may argue that a key concern would by whether and how an individual may be capable of monitoring her thoughts and perceptions and how those may be affected by opiate addiction. Understanding the relationships between metacognition in these tasks and the real-life experiences could introduce new methods for the diagnosis and treatment of patients with self-awareness deficits such as addiction.

## Methods

### Participants

Participants consisted of a group of twenty five Substance Dependent Individuals (SDI) and a matched control group of twenty five healthy individuals. Two of the SDIs and one of the control participants were excluded from the analysis due to outlier accuracy level in the perceptual task. Other participants (SDI N = 23, Control N = 24) successfully finished both tasks. The study was approved by the ethics board of the Tehran University of Medical Sciences (TUMS) and the experiment was carried out in accordance with the Declaration of Helsinki.

All participants in the study were male, Persian native speaker, aged between 25 to 50 years, with at least 11 years of formal education and normal or corrected to normal visual acuity. Other eligibility requirements included having no history of neurological disorders or psychiatric disorders based on DSM IV criteria (except those directly related to DSM IV diagnosis of past opiate dependence). All participants gave informed consent for participation.

SDIs were recruited from the research clinic at Iranian National Center for Addiction Studies (INCAS) who were stable on Methadone Maintenance Treatment for at least 3 months. The treatment program at INCAS requires patients to take routine urine drug-screening tests on a biweekly basis to confirm adherence to treatment and abstinence from any drug abuse except Nicotine. Patients with background of positive urine test during the last 3 months were not included in the study. Average weekly methadone dose was 121.3 mg (SD = 45.2, range 40–250) and mean duration of enrollment in the treatment program was 2.73 years (SD = 2.46, range 0.25–7.92). Control individuals were volunteers recruited from the staff of University of Tehran and Imam Khomeini hospital. They had no self-reported history of drug dependence (except to Nicotine). Tables 3 and 4 represent demographics of participants and self-reported drug use history of the SDIs. There were no significant differences between the SDIs and control participants in mean age or education.

**Table 3:** Demographics (mean age and years of education) for participants in the SDI and control group.

	Mean (SD)	
	SDI (N = 23)	Control (N = 24)
**Age**	37.52 (6.31)	34.09 (6.85)
**Education (years)**	12.96 (1.72)	12.91 (1.66)

**Table 4:** Self-reported drug use history of SDIs.

	Lifetime abuse/dependence	Main drug of abuse when treatment started
**Opium**	87%	48%
**Brown Heroin**	52%	43%
**Crystalline White Heroin**	43%	9%
**Methamphetamine**	35%	—
**Cannabis**	39%	—
**Alcohol**	17%	—

### Tasks and procedures

All participants performed a perceptual and a memory task. Both tasks were computerized, programmed in MATLAB (Mathworks Inc.) using COGENT 2000 toolbox (http://www.vislab.ucl.ac.uk/cogent.php). Order of tasks was counterbalanced among participants to eliminate the potential effect of task order on the results.

The perceptual task was adopted from Fleming, *et al*.^31^ and Song, *et al*.^32^. In a 2-AFC task, two stimulus displays were presented in successive time intervals in each trial. Each visual stimulus display consisted of six Gabor gratings placed around a central fixation point (eccentricity of Gabor center: 3.6 visual degrees, 3.2 cm). Each Gabor patch subtended 1.5 degrees (1.3 cm) and consisted of horizontal alternating light and dark bars modulated at a spatial frequency of 2.2 cycles per visual degree with a contrast of 20%. The perceptual task was performed in a dark room where the monitor was the only source of light. On each trial of the task, two stimuli were presented on the screen serially, each for 85 msec, with an ISI of 1000 msec. One grating in one of the stimulus displays (target stimulus) had a higher contrast than the rest of the gratings. The interval in which the contrast oddball was displayed and its spatial location among the 6 Gabor gratings in the target stimulus was assigned randomly across trials. The contrast of the oddball was determined using a 1-up 2-down staircase procedure that was expected to converge to an approximate accuracy of 71% for each participant. At the beginning of the perceptual experiment, the contrast of the oddball was set to 20% higher than the distractors and it changed adaptively according to participant’s performance. The oddball contrast was increased by a step after one incorrect response or decreased by a step after two consecutive correct responses. Step size was set to 3%.

Followed by the stimulus presentation on each trial, the participant indicated his 2-alternatvie choice whether the target stimuli was in the first or the second interval. Next, he rated his confidence about the accuracy of the response using numbers 1 (very unsure) to 6 (very sure).The perceptual task consisted of 180 trials (two blocks of 90-trials). A variable number of practice trials (between 5 and 25, depending on when the participant indicated to have completely understood the task procedure) were conducted prior to the main task.

Memory items were Farsi words chosen from Persian Linguistic Database (PLDB)^33^ a comprehensive database of Persian language words. Nouns with 4 or 5 letters, 2 syllables and word frequency between 6 and 150 per million were selected from PLDB. After excluding words with emotional valence and those with drug-related meaning, 290 words were chosen.

We also controlled for words’ imageability in order to investigate the influence of imageability on metacognitive ability for verbal memory. Imageability of a word is a measure of how quickly and easily it invokes a mental image. For instance, according to most people, the word “tree” is highly imageable, and the word “result” is less so. We ran a survey to select the most and the least imageable words from the list of 290 words. For this purpose, words were rated for imageability by 50 university students who did not participate in the main experiment. Volunteers were provided standardized instructions on imageability rating. The rating was on a 7-point scale with number 1 indicating “very low imageability” to 7 for “very high imageability”. Each volunteer rated a variable number of words, between 96 to all 290, and finally each word was rated by 19 volunteers. Ratings were averaged across raters and words were sorted by mean imageability. The 100 words with the highest and 100 with the lowest mean imageability were selected as the final list of 200 words.

The memory task followed similar protocol to that of Baird, *et al*.^24^. We employed a word recognition memory test consisting of a learning and a recognition phase. During the learning phase, 100 words were presented on the screen, one at a time, half of them selected from the high imageable set and the other half from the low imageable set. The list of 100 words were randomly chosen for each participant from the pool of 200 words (50 from 100 high imageable and 50 from 100 low imageable). Each word was displayed at the center of the screen for 1500 msec, with an ISI of 1000 msec between subsequent words. A fixation point was shown on the center of display during the ISI. In the recognition phase, all 200 words were presented to the participant one at a time. The participant decided if the word was new or had previously been presented in the learning phase. They then indicated their confidence rating about the response accuracy on a 6-point scale (similar to the perceptual task).

**Figure 3:**
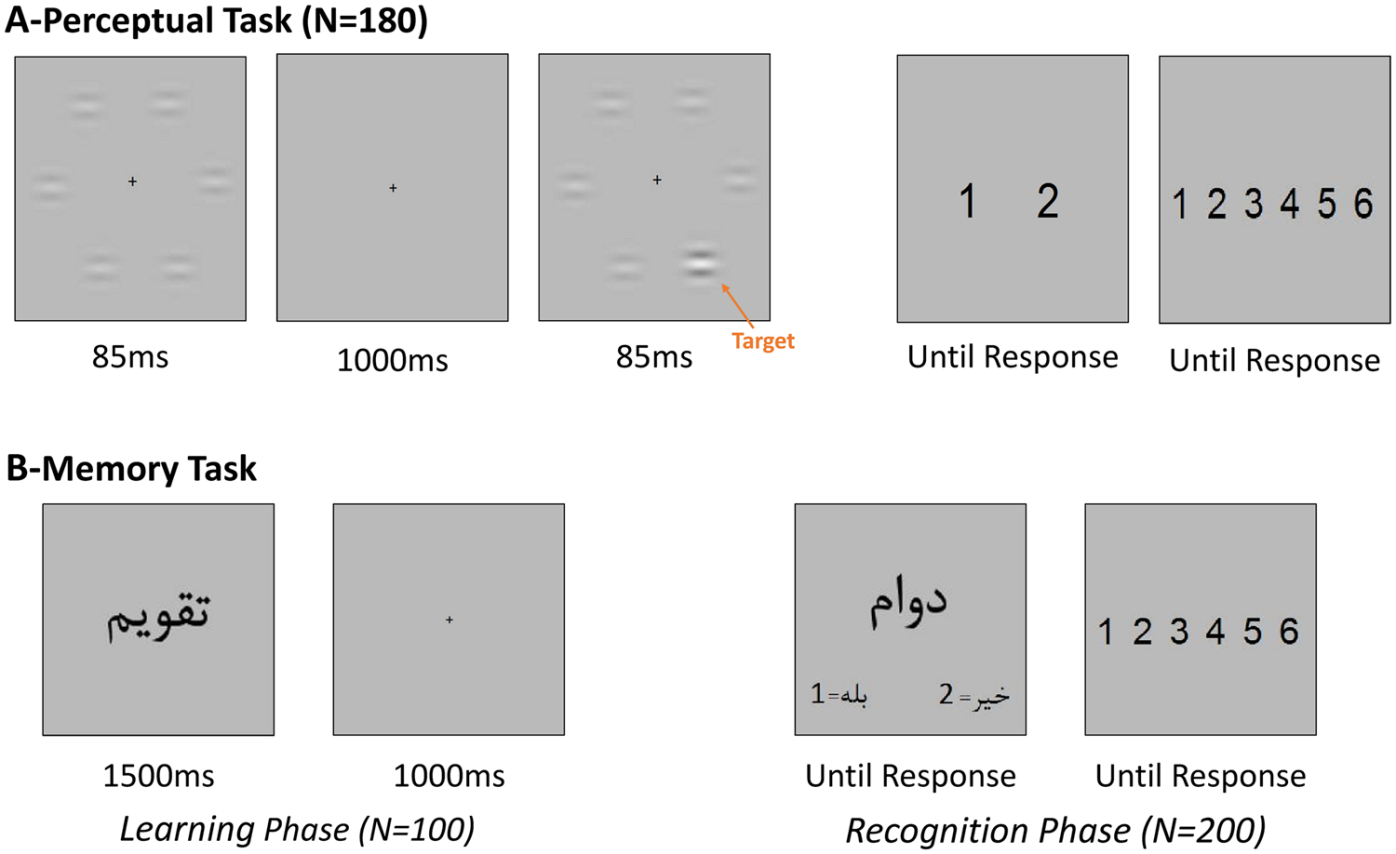
Outline of the perceptual (**A**) and memory (**B**) tasks. 2-AFC responses in both tasks are followed by rating confidence in a 1–6 scale. (**A**) One trial in the perceptual task (N = 180) consisted of two displays of 6 Gabor gratings each presented for 85 msec and separated by a 1000 msec ISI. Participant should indicate whether he observed the odd Gabor in the 1st or the 2nd interval. (**B**) The memory task included a learning and a recognition phase. During the learning phase (N = 100) each word is displayed for 1500 msec with 1000 msec interval between subsequent words. In the recognition phase (N = 200) a word was presented asking whether it was already seen or not.

In both tasks, participants responded using the keyboard, with no time limitation. They were instructed to enter the 2-AFC responses by pressing numbers 1 (indicating the 1^st^ interval in the perceptual task and “Old” in the memory task) and 2 on the keypad with left hand, and rate their confidence by numbers 1 to 6 on the number pad with right hand. Participants did not receive any feedback about their performance. Figure 3 illustrates the outline of the perceptual and memory tasks.

### Statistical Analysis

Intuitively, better self-monitoring is associated with higher confidence when making correct decisions and lower confidence when incorrect. However comparing raw values of mean confidence between people can be misleading because the effect of individual biases such as general over- or under-confidence could be overlooked. An ideal measure of metacognition should be independent of response and confidence biases. In other words, it should discriminate a person’s true metacognitive sensitivity from her inclination towards selecting one alternative over the other, or some numbers for rating confidence over the others. Furthermore, a good measure of metacognition should manifest pure introspective ability independent of one’s first order performance.

Signal Detection Theory (SDT)^21^ provides a framework to obtain an estimate of metacognition with such characteristics. Meta-d′ is a measure based on the SDT which has recently been developed to estimate metacognition independent of performance or response bias^22, 23, 34^. The main theoretical assumption behind the meta-d′ approach is that the choice and the confidence ratings originate from two different SDT models called type 1 and type 2 SDT each with their own respective sensitivity parameters. Metacognitive ability is the ratio of the type 2 sensitivity to type 1 sensitivity, implying how much information is available to the observer at the meta-level, compared to the object level. For the perceptual task, we used the M-ratio = meta-d′/d′ as the measure of relative type 2 sensitivity to account for metacognitive ability.

Computing metacognition in the memory task however, is more challenging. The ‘old’-‘new’ stimuli aren’t necessarily modeled by equal variance SDT, so we used unequal variance SDT model of meta-d′. For this purpose, the relative variance of the SDT model probability distributions are calculated based on the participant’s responses and confidence ratings, and assumed as a constant parameter in both type 1 and type 2 SDT models. Then, the unequal variance version of M-ratio would be M-ratio = meta-da/da. We used this measure to compute memory task metacognition. One caveat of this approach is that the variance is calculated using both type 1 and type 2 data, and is supposed to be equal in both SDT models. This challenges the presumption that type 1 and type 2 SDT models are independent (see Maniscalco and Lau^23^ for more details). We also calculated Equal-variance meta-d′ for memory task metacognition and rechecked our results. We used the Matlab code available at http://www.columbia.edu/~bsm2105/type2sdt/ for computing meta-d′ and meta-da in perceptual and memory tasks.

Pearson linear correlation coefficient was used to investigate relations between variables. Main effects and interaction effect of group and task on a measure was examined using mixed effect Group(SDI/control) × Task(perceptual/memory) Analysis of Variance (ANOVA). The statistical test used for comparing means was two-tailed t-test, and the assumption of equality or Inequality of variance was made according to Levene’s Test. Significance threshold (α) for all statistical tests was set to 0.05.
